# Effects of Initiation Age of Starter Feeding on Growth Performance, Immunity and Antioxidant Capacity, Gastrointestinal Development, and Microbial Communities in Suckling Lambs

**DOI:** 10.3390/microorganisms13112490

**Published:** 2025-10-30

**Authors:** Shaoyang Pang, Xiangjian Peng, Shu Li, Junli Niu, Wenqi Wang, Yanfeng Liu, Cunxi Nie, Wenju Zhang

**Affiliations:** 1 College of Animal Science and Technology, Shihezi University, Shihezi 832061, China; 15251239380@163.com (S.P.); pxj1016pxj@sina.com (X.P.); lishu00520@163.com (S.L.); niujunliaa@126.com (J.N.); 2 Feed Research Institute, Xinjiang Academy of Animal Science, Urumqi 830011, China; xjslswwq@163.com (W.W.); xjslslyf@163.com (Y.L.)

**Keywords:** gastrointestinal development, growth performance, immunity and antioxidant capacity, lambs, microbial community, rumen fermentation

## Abstract

The optimal timing for initiating starter feeding in lambs remains controversial, warranting a systematic evaluation of its effects across multiple indicators. This study investigated the effects of initiating starter feeding at 7, 14, or 21 days of age on growth performance, rumen fermentation, serum immunity, gastrointestinal development, and microbiota in Hu lambs. Forty-five newborn lambs were randomly assigned to three groups (*n* = 15 per group) and fed starter until slaughter at weaning (60 days). The 14-day group exhibited significantly higher body weight (BW) at 49 and 60 days compared with the 7-day group (*p* < 0.05), and greater average daily gain (ADG) during 28–35 days than the 21-day group. Rumen ammonia nitrogen (NH_3_-N), acetate, propionate, valerate, and total volatile fatty acids (VFAs) were higher in the 7-day and 14-day groups than in the 21-day group (*p* < 0.05). The 14-day group showed lower pro-inflammatory cytokines (IL-1β, TNF-α) and higher immunoglobulins (IgA, IgG, IgM) and anti-inflammatory cytokines (IL-2, IL-4) (*p* < 0.05). This group also displayed improved rumen papilla width, jejunal villus dimensions, and reduced crypt depth. Beneficial microbes such as Christensenellaceae_R-7_group and Butyrivibrio were enriched in the 14-day and 21-day groups. In conclusion, initiating starter feeding at 14 days of age optimizes growth, rumen function, immune response, and colonization of beneficial microbiota in Hu lambs.

## 1. Introduction

The early growth and development stage of lambs is crucial for determining their lifelong production performance [1]. During this phase, although breast milk is the primary source of nutrition, the milk yield of ewes gradually decreases as lambs grow older, making it challenging to meet the nutritional demands of the rapidly growing lambs [2]. Thus, initiating supplemental feeding with starter feed at an appropriate time has become a core strategy to bridge the nutritional gap, promote rumen development, and mitigate weaning stress [3]. Reasonable use of starter feed not only enhances the growth rate and feed conversion efficiency of lambs [4,5], but also improves their digestive function by regulating the early colonization of rumen microbiota, thereby laying a solid foundation for the subsequent fattening stage [6,7]. Nevertheless, the optimal timing for initiating supplemental feeding remains a contentious issue in current lamb rearing management. Initiating supplemental feeding too early may result in low utilization efficiency of starter feed due to the immature feeding ability of lambs; conversely, starting it too late may cause the loss of the “window period” for rumen development, leading to post-weaning growth retardation [8].

In recent years, despite progress in research on the age at which to begin supplemental feeding, findings remain inconsistent. Some studies indicate that initiating starter feeding 7–10 days after birth significantly increases weaning weight [8], while other studies suggest that supplemental feeding should be started after 14 days of age to match the maturation of rumen morphology and function [4]. However, a systematic comparison of the effects of different feeding initiation times on multidimensional indicators, including growth, rumen development, immune status, and microbial colonization, under the same husbandry conditions is still lacking.

Xinjiang Hu sheep are a high-yielding meat breed characterized by early sexual maturity, year-round estrus cycles, and adaptability to intensive farming systems. Known for their high reproductive efficiency, they represent a valuable economic resource for commercial producers. Considering the breed’s economic importance, the regional advantages of Xinjiang, and existing research gaps, we hypothesized that introducing starter feed at 14 days of age would optimize rumen development, enhance immune function, and promote beneficial microbial colonization, thereby improving overall growth performance in suckling Hu lambs. To test this hypothesis, we systematically evaluated the effects of initiating supplementation at 7, 14, and 21 days of age on growth performance, rumen fermentation, serum immunity, gastrointestinal development, and microbial communities.

## 2. Materials and Methods

### 2.1. Experimental Animals

The animals were provided by Yitianjun Animal Husbandry Branch of Xinjiang Gongnaisi Breeding Sheep Farm Co., Ltd. (Geographic coordinates: [83°37′ E], [43°09′ N], Gongnaisi, China). The animal care and use protocol for this study was approved by the Animal Welfare Committee of Shihezi University (Shihezi, China) under the ethical approval code A2025-852. The feeding management strictly followed the normal procedures of the breeding company, and all lambs received standardized immunization protocols. The experimental site and related equipment were sterilized accordingly.

### 2.2. Experimental Design and Feeding Management

A total of 45 newborn singleton male Hu lambs with similar birth weights (3.89 ± 0.98 kg) were selected and randomly assigned to three groups (*n* = 15 per group): the 7 d, 14 d, and 21 d groups, based on the age at which starter feeding was initiated. Lambs in the 7 d, 14 d, and 21 d groups began receiving the starter diet at 7, 14, and 21 days of age, respectively. The composition and nutritional content of the starter feed are provided in Table 1. Throughout the trial, starter feed was provided daily to ensure refusals remained available. Lambs were housed separately from their dams in pens. In addition to the starter feed, all lambs were allowed to suckle their dams three times per day (08:00–10:00, 14:00–16:00, and 19:00–21:00) and had free access to drinking water. Supplemental starter feeding continued until all lambs were weaned at 60 days of age.

### 2.3. Sample Collection

At the beginning of the experiment, each lamb was weighed, with subsequent body weight (BW) measurements performed weekly. When the lambs reached 60 days of age, five lambs from each group were randomly selected and fasted for 12 h. Venous blood (10 mL) was then collected from each selected lamb. After standing for 30 min, the whole blood was centrifuged at 3000× *g* for 15 min to isolate serum. The lambs were then euthanized via exsanguination from the jugular vein. Dissection was performed immediately under sterile conditions. The rumen was isolated, and ruminal fluid was collected, filtered through four layers of sterile gauze, aliquoted into 2 mL cryovials, and stored at −80 °C. Tissue samples from the rumen, duodenum, and jejunum were collected, rinsed thoroughly with physiological saline, and fixed in 4% paraformaldehyde solution. Finally, the contents of the duodenum and jejunum were collected, placed into sterile cryovials, immediately frozen in liquid nitrogen, and stored at −80 °C for subsequent 16S rRNA sequencing and microbial community analysis.

#### 2.3.1. Nutrient Content of the Starter Diet

The crude protein and crude fat contents in the starter feed were determined according to Eghtedari et al. [9], using a Kjeldahl analyzer (VELP Scientifica, Usmate, Italy) and an automatic Soxhlet extraction system (AntiTeck, Guangzhou, China), respectively. Meanwhile, the calcium (Ca) and phosphorus (P) contents were analyzed following Moreira et al. [10] by flame atomic absorption spectrometry (Analyst 200, PerkinElmer Inc., Waltham, MA, USA). The contents of neutral detergent fiber (NDF) and acid detergent fiber (ADF) were measured using the methods proposed by Van Soest et al. [11].

#### 2.3.2. Serum Immunity and Antioxidant Capacity

Serum levels of catalase (CAT), malondialdehyde (MDA), superoxide dismutase (SOD), total antioxidant capacity (T-AOC), interleukin-1β (IL-1β), interleukin-2 (IL-2), interleukin-4 (IL-4), immunoglobulin A (IgA), immunoglobulin G (IgG), immunoglobulin M (IgM), and tumor necrosis factor-α (TNF-α) were strictly measured in accordance with the instructions of the assay kits provided by Shanghai Enzyme-linked Biotechnology Co., Ltd. (Shanghai, China) [12].

#### 2.3.3. Rumen Fermentation Parameters

pH was measured using a portable pH tester (PH 800, SMART SENSOR, Hong Kong, China). The quantification of VFAs was performed according to the method established by Li et al. (2025) [13]. Briefly, thawed rumen fluid was initially centrifuged (3000× *g*, 4 °C, 10 min) to remove particulate matter. Subsequently, 1 mL of the resulting supernatant was mixed with 200 μL of 25% (*w*/*v*) meta-phosphoric acid to precipitate proteins. The mixture was then centrifuged again (10,000× *g*, 4 °C, 10 min), and the supernatant was filtered through a microporous membrane for purification. Finally, the VFA concentrations were determined using a gas chromatography system (7890B, Agilent, Santa Clara, CA, USA). The GC conditions were as follows: the injector temperature was 250 °C, and the transfer line temperature was set at 260 °C. The FID detector was maintained at 300 °C. The oven temperature program was set as follows: 80 °C for 1 min, increased to 200 °C at a rate of 20 °C/min, and held for 2 min. Nitrogen was used as the carrier gas at 1.0 mL/min. For headspace analysis, 10 μL of the vapor phase from the 1 mL quantitative loop was injected into the GC inlet. Solid samples were dried and sieved through a 60-mesh sieve to ensure homogeneity.

#### 2.3.4. Gastrointestinal Tissue Sectioning and Related Growth Genes

Rumen and intestinal tissue sections were processed according to the method described by Zhao et al. [14]. After fixation in 4% paraformaldehyde for 24–48 h, the tissues were routinely processed into paraffin-embedded blocks following standard histological procedures. Sections of 4–5 μm thickness were prepared and stained with hematoxylin and eosin (H&E) [15]. Images were captured using an upright optical microscope (Nikon, Tokyo, Japan), and parameters such as villus length were measured with ImageJ 2.3.0 software (Media Cybernetics, Bethesda, MD, USA).

Following extraction of total RNA using TRIzol reagent (TransGen Biotech, Beijing, China) and spectrophotometric assessment of its purity/concentration on a NanoDrop instrument (Thermo Fisher Scientific, Waltham, MA, USA), first-strand cDNA synthesis was performed with a reverse transcription kit (Master Premix, Beijing, China). Quantitative real-time polymerase chain reaction (qRT-PCR) analysis was then carried out on a Roche LightCycler 96 system (Basel, Switzerland) in a 20 μL reaction volume containing 15 μL PerfectStart Green qPCR SuperMix (TransGen Biotech, Beijing, China), 10 ng cDNA, and 0.2 μM of each primer. The protocol involved initial denaturation (95 °C, 30 s) and 50 cycles of denaturation (95 °C, 10 s), annealing (60 °C, 15 s), and extension (72 °C, 10 s) [16]. Each assay was run in triplicate, and the 2^−ΔΔCt^ method was applied to quantify relative gene expression [17]. He primer sequences for target genes are provided in Table 2, with *β-actin* serving as the endogenous reference gene.

#### 2.3.5. 16S rRNA Microbial Sequencing Analysis

Following extraction of ruminal and intestinal contents with the E.Z.N.A.^®^ Microbiome DNA Kit (Omega Bio-tek, Norcross, GA, USA), the quality and concentration of the genomic DNA were evaluated via electrophoresis and a NanoDrop2000 instrument (Thermo Fisher Scientific, Waltham, MA, USA). The V3–V4 region of the 16S rRNA gene was amplified with barcoded primers 338F/806R using TransStart Fastpfu DNA Polymerase (TransGen Biotech, Beijing, China) under the following polymerase chain reaction (PCR) conditions: 95 °C for 3 min; 28 cycles of 95 °C for 30 s, 55 °C for 30 s, and 72 °C for 45 s; and 72 °C for 10 min [18].

Amplified products were purified with a gel extraction kit and quantified using Qubit 4.0. Sequencing libraries were prepared with the NEXTFLEX Rapid DNA-Seq Kit through adapter ligation [19], bead-based purification, and enrichment PCR. The libraries were sequenced on the Illumina Nextseq2000 platform (Majorbio, Shanghai, China).

Bioinformatic analyses including taxonomic assignment, alpha and beta diversity, and database comparisons were performed using QIIME 1.91, Mothur 1.30.2, and the SILVA 138 database on the Majorbio Cloud Platform [20]. Raw sequencing data are deposited in the NCBI SRA under accession PRJNA1326499.

### 2.4. Statistical Analysis

The experimental data were analyzed using SPSS 20.0. BW data were subjected to repeated measures ANOVA in a completely randomized design. The model included fixed effects of treatment (initiation age), time (days of age), and their interaction, with individual lamb as a random effect:

Yijk = μ + τi + βj + (τβ)ij + γk + εijk

where Yijk represents the observation for the k-th lamb in the i-th treatment at the j-th time point, μ is the overall mean, τi is the treatment effect, βj is the time effect, (τβ)ij is the interaction effect, γk is the random lamb effect, and εijk is the residual error.

Mauchly’s test verified sphericity. Significant interactions (*p* < 0.05) were followed by simple effects analysis. Average daily gain (ADG) data were analyzed by one-way ANOVA within each period. Group differences were assessed using Duncan’s test (*p* < 0.05). Additionally, Spearman’s rank correlation analysis was performed to assess relationships between rumen microbiota and host parameters, with results visualized as a clustered heatmap.

## 3. Results

### 3.1. Effects of Starter Feeding Initiation Age on Growth Performance of Suckling Lambs

As presented in Table 3, a significant treatment × time interaction was observed for BW (*p* < 0.05). At 49 and 60 days of age, the BW of lambs in the 14 d and 21 d groups was significantly higher than that in the 7 d group (*p* < 0.05). Regarding ADG, during the 28–35 days period, the ADG of lambs in the 7 d and 14 d groups was significantly higher than that in the 21 d group (*p* < 0.05).

### 3.2. Effects of Starter Feeding Initiation Age on Rumen Fermentation Parameters of Suckling Lambs

As shown in Table 4, in the 7 d and 14 d lamb groups, the rumen fluid had significantly higher concentrations of ammonia nitrogen (NH_3_-N), acetate, propionate, and total volatile fatty acids (TVFA) than that in the 21 d group (*p* < 0.05). The valerate content in the rumen fluid of lambs in the 14 d group was significantly higher than that in the 21 d group (*p* < 0.05). In addition, the concentrations of other VFAs explored, including isobutyric, butyric, and isovaleric acids, were not significantly different among the three groups (*p* > 0.05).

### 3.3. Effects of Starter Feeding Initiation Age on Serum Immunity and Antioxidant Capacity of Suckling Lambs

As shown in Table 5, the initiation time of starter feed had no significant influence on the serum antioxidant capacity of lambs (*p* > 0.05), but exerted a significant effect on the serum immune indicators (*p* < 0.05). The serum MDA contents of lambs in the 14 d group and 21 d group were significantly lower than that in the 7 d group (*p* < 0.05). Compared with the 7 d group, the 14 d group had significantly lower serum levels of IL-1β and TNF-α, while significantly higher contents of IL-2, IL-4, IgA, IgG, and IgM (*p* < 0.05). Additionally, the serum TNF-α level in the 21 d group was significantly higher than that in the 14 d group (*p* < 0.05).

### 3.4. Effects of Starter Feeding Initiation Age on Gastrointestinal Tract Development of Suckling Lambs

As shown in Table 6, the expression level of the ruminal epithelial gene *TGFβ1* in the 14 d group was significantly higher than that in the 7 d group (*p* < 0.05). Although no significant differences were observed in the expression of other genes, the expression levels of gastrointestinal growth-related genes in the 14 d group showed an increasing trend compared to both the 7 d and 21 d groups, but these differences did not reach statistical significance (*p* > 0.05).

As shown in Table 7, starter feeding initiation age differentially influenced gastrointestinal morphology. In the rumen, papilla width was greater in the 7 d and 14 d groups than in the 21 d group (*p* < 0.05), whereas papilla length and muscularis thickness were unaffected (*p* > 0.05). Duodenal morphology (villus height, villus width, crypt depth, muscularis thickness, V/C ratio) was not significantly altered (*p* > 0.05). In the jejunum, however, both the 14 d and 21 d groups exhibited significantly reduced villus height, villus width, and crypt depth compared to the 7 d group (*p* < 0.0). Jejunal muscularis thickness and V/C ratio were similar across all groups (*p* > 0.05).

### 3.5. Effects of Starter Feeding Initiation Age on Rumen Microbial Communities of Suckling Lambs

**Figure 1:**
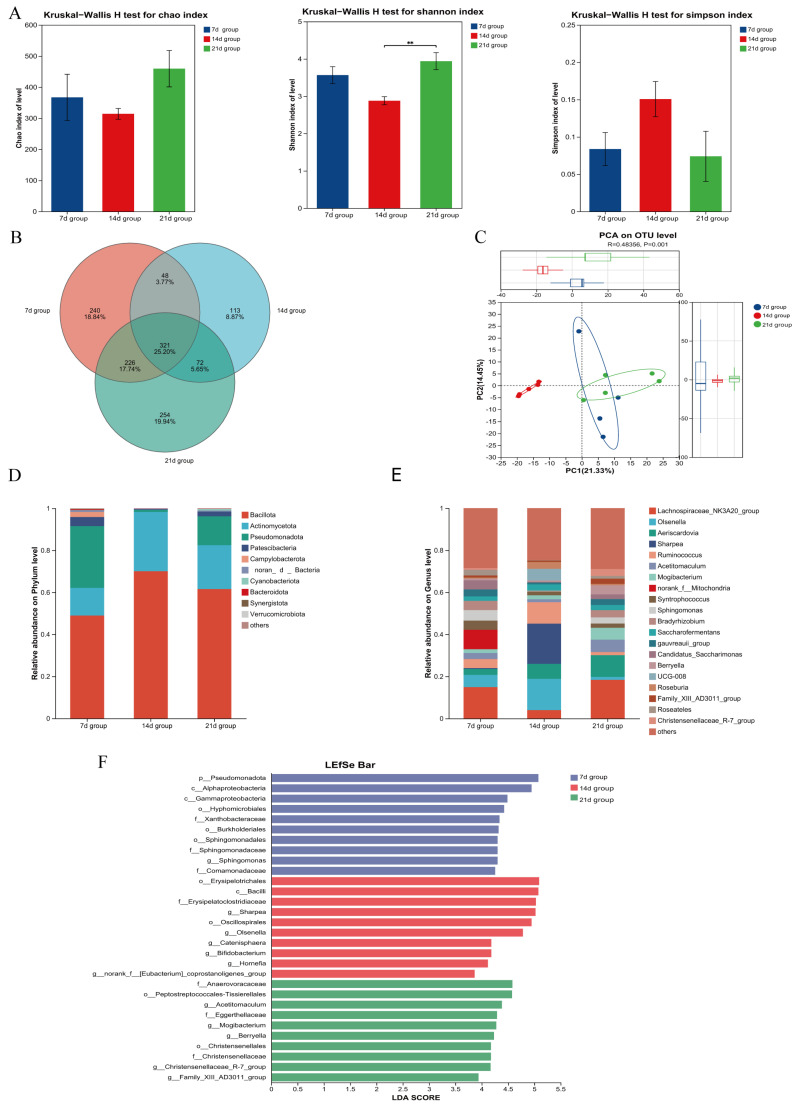
Effects of Initiating Starter Feeding at Different Ages on Rumen Microbiota of Lambs. (**A**) Assessment of microbial richness and evenness using α-Diversity indices (Chao, Shannon, and Simpson). (**B**) Venn diagram illustrating the shared and unique operational taxonomic units (OTUs) among experimental groups. (**C**) β-Diversity and inter-group differences displayed through Principal Component Analysis (PCA). (**D**) Compositional profile of microbial communities at the phylum level. (**E**) Relative abundance of microbial taxa at the genus level. (**F**) Differentially abundant taxa across groups identified by linear discriminant analysis effect size (LEfSe). Statistical significance was defined as *p* < 0.01 (**).

As shown in Figure 1A, the Shannon and Chao indices of rumen microbiota in the 7 d and 21 d groups were significantly higher than those in the 14 d group, while the Simpson index was significantly lower (*p* < 0.05). The numbers of unique operational taxonomic units (OTUs) in the 7 d and 21 d groups were 700 and 1158, respectively, both higher than that in the 14 d group (530) (Figure 1B).

Beta diversity analysis demonstrated a distinct separation between the 7 d group and the 14 d and 21 d groups, indicating significant differences in microbial community composition (*p* < 0.05) (Figure 1C).

Analysis at the phylum level showed that the top two dominant phyla in the 7 d, 14 d, and 21 d groups were *Bacillota* and *Bacteroidota* (Figure 1D). Linear discriminant analysis Effect Size (LEfSe) analysis further indicated that the relative abundances of *Bacillota* and *Patescibacteria* were significantly higher in the 7 d and 21 d groups than in the 14 d group, while the abundance of *Pseudomonadota* was significantly lower in the 14 d group (*p* < 0.05) (Figure 1F).

At the genus level, the top two dominant genera in the 7 d group were *Xylanibacter* and *norank_f__F082*, whereas those in the 14 d and 21 d groups were *Xylanibacter* and *Rikenellaceae_RC9_gut_group* (Figure 1E). Using LEfSe analysis to identify differentially abundant taxa, we found that the 14 d group had significantly lower abundances of *Christensenellaceae_R-7_group*, *Candidatus_Saccharimonas*, *Butyrivibrio*, *norank_f__UCG-010*, and *Fretibacterium* compared to the 7 d and 21 d groups (*p* < 0.05) (Figure 1F).

### 3.6. Effects of Starter Feeding Initiation Age on Small Intestinal Microbial Communities of Suckling Lambs

#### 3.6.1. Effects on Duodenal Microbial Communities

**Figure 2:**
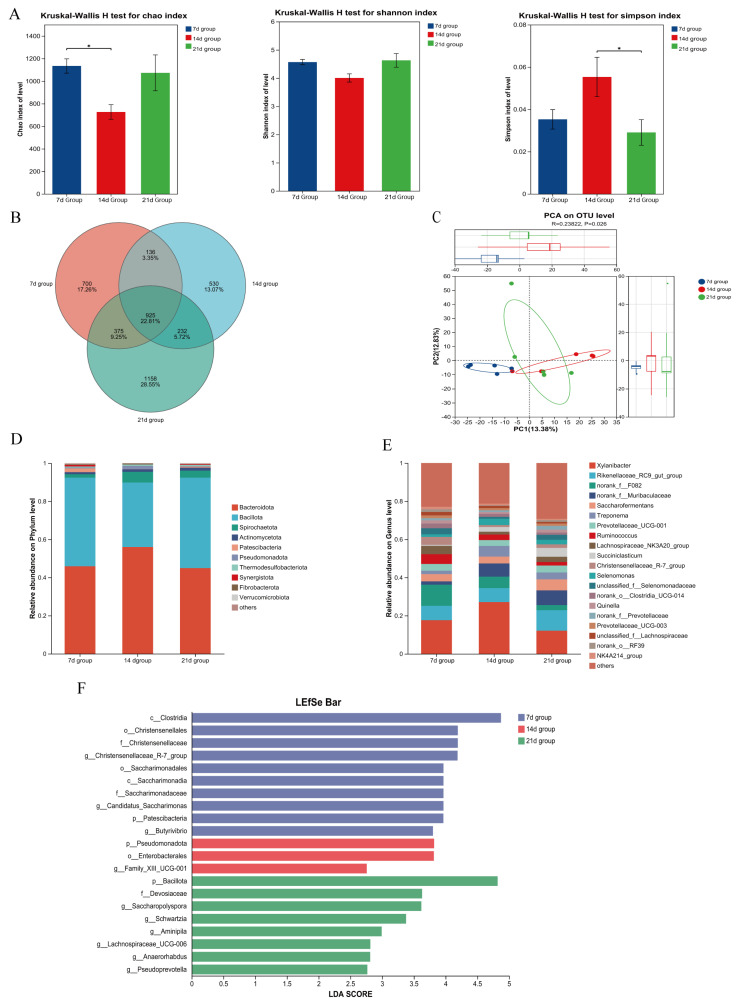
Effects of Initiating Starter Feeding at Different Ages on Duodenal Microbiota of Lambs. (**A**) Assessment of microbial richness and evenness using α-Diversity indices (Chao, Shannon, and Simpson). (**B**) Venn diagram illustrating the shared and unique operational taxonomic units (OTUs) among experimental groups. (**C**) β-Diversity and inter-group differences displayed through Principal Component Analysis (PCA). (**D**) Compositional profile of microbial communities at the phylum level. (**E**) Relative abundance of microbial taxa at the genus level. (**F**) Differentially abundant taxa across groups identified by linear discriminant analysis effect size (LEfSe). Statistical significance was defined as *p* < 0.05 (*).

As shown in Figure 2A, the Shannon index in the 14 d group was significantly lower than that in the 21 d group (*p* < 0.05), while no significant differences were observed in the Simpson index or Chao1 value (*p* > 0.05). The numbers of unique OTUs in the 7 d and 21 d groups were 240 and 254, respectively, both higher than that in the 14 d group (113) (Figure 2B). Beta diversity analysis revealed complete separation of the 14 d group from both the 7 d and 21 d groups, indicating significant differences in microbial community composition (*p* < 0.05) (Figure 2C).

Analysis at the phylum level showed that the top two dominant phyla in the 7 d group were *Bacillota* and *Pseudomonadota*; in the 14 d group, *Bacillota* and *Actinomycetota*; and in the 21 d group, *Bacillota* and *Actinomycetota* (Figure 2D). LEfSe analysis indicated that the abundances of *Olsenella* and *Sharpea* were significantly higher in the 14 d group than in the 7 d and 21 d groups (*p* < 0.05), while the abundances of *Acetitomaculum* and *Mogibacterium* were significantly higher in the 21 d group than in the 14 d group (*p* < 0.05) (Figure 2F).

At the genus level, the top two dominant genera in the 7 d group were *Lachnospiraceae_NK3A20_group* and *norank_f__Mitochondria*; in the 14 d group, *Sharpea* and *Olsenella*; and in the 21 d group, *Lachnospiraceae_NK3A20_group* and *Aeriscardovia* (Figure 2E). Further LEfSe analysis demonstrated that the relative abundances of *Olsenella*, *Sharpea*, and *UCG-008* in the 14 d group were significantly higher than those in the 7 d and 21 d groups (*p* < 0.05), while the relative abundances of *Sphingomonas* and *Bradyrhizobium* were significantly lower (*p* < 0.05). The relative abundances of *Acetitomaculum* and *Mogibacterium* in the 21 d group were significantly higher than those in the 14 d group (*p* < 0.05) (Figure 2F).

#### 3.6.2. Effects of Starter Feeding Initiation Age on Jejunal Microbial Communities of Suckling Lambs

**Figure 3:**
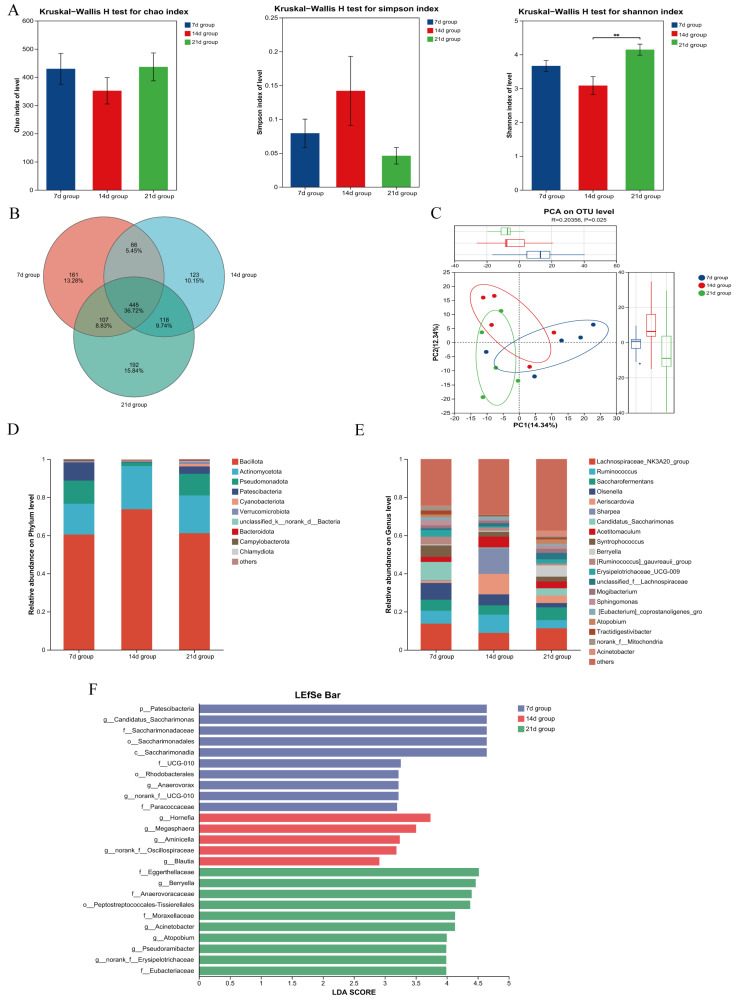
Effects of Initiating Starter Feeding at Different Ages on Jejunal Microbiota of Lambs. (**A**) Assessment of microbial richness and evenness using α-Diversity indices (Chao, Shannon, and Simpson). (**B**) Venn diagram illustrating the shared and unique operational taxonomic units (OTUs) among experimental groups. (**C**) β-Diversity and inter-group differences displayed through Principal Component Analysis (PCA). (**D**) Compositional profile of microbial communities at the phylum level. (**E**) Relative abundance of microbial taxa at the genus level. (**F**) Differentially abundant taxa across groups identified by linear discriminant analysis effect size (LEfSe). Statistical significance was defined as *p* < 0.01 (**).

As shown in Figure 3A, the Shannon index in the 21 d group was significantly higher than that in the 14 d group (*p* < 0.05), while no significant differences were observed in the Chao1 value or Simpson index (*p* > 0.05), although the Simpson index exhibited a linear trend. The numbers of unique OTUs in the 7 d and 21 d groups were 161 and 192, respectively, both higher than that in the 14 d group (123) (Figure 3B). Beta diversity analysis revealed clear separation among the three groups, indicating significant differences in microbial community composition (*p* < 0.05) (Figure 3C).

Analysis at the phylum level showed that the top two dominant phyla in the 7 d, 14 d, and 21 d groups were *Bacillota* and *Actinomycetota* (Figure 3D). LEfSe analysis further indicated that the relative abundances of *Patescibacteria* and *Verrucomicrobiota* in the 7 d and 21 d groups were significantly higher than those in the 14 d group (*p* < 0.05) (Figure 3F).

At the genus level, the top two dominant genera in the 7 d group were *Lachnospiraceae_NK3A20_group* and *Candidatus_Saccharimonas*; in the 14 d group, *Sharpea* and *Aeriscardovia*; and in the 21 d group, *Lachnospiraceae_NK3A20_group* and *Saccharofermentans* (Figure 3E). Further statistical analysis demonstrated that the relative abundances of *Candidatus_Saccharimonas*, *Atopobium*, and *norank_f__Erysipelotrichaceae* in the 7 d and 21 d groups were significantly higher than those in the 14 d group (*p* < 0.05), while the relative abundances of *UCG-008* and Hornefia were significantly lower (*p* < 0.05). The relative abundances of *Berryella*, *Acinetobacter*, and *norank_f__Erysipelotrichaceae* in the 21 d group were significantly higher than those in the 7 d and 14 d groups (*p* < 0.05) (Figure 3F).

### 3.7. Correlation Analysis Between Rumen Microbiota and Rumen Fermentation Parameters/Immune Indicators

**Figure 4:**
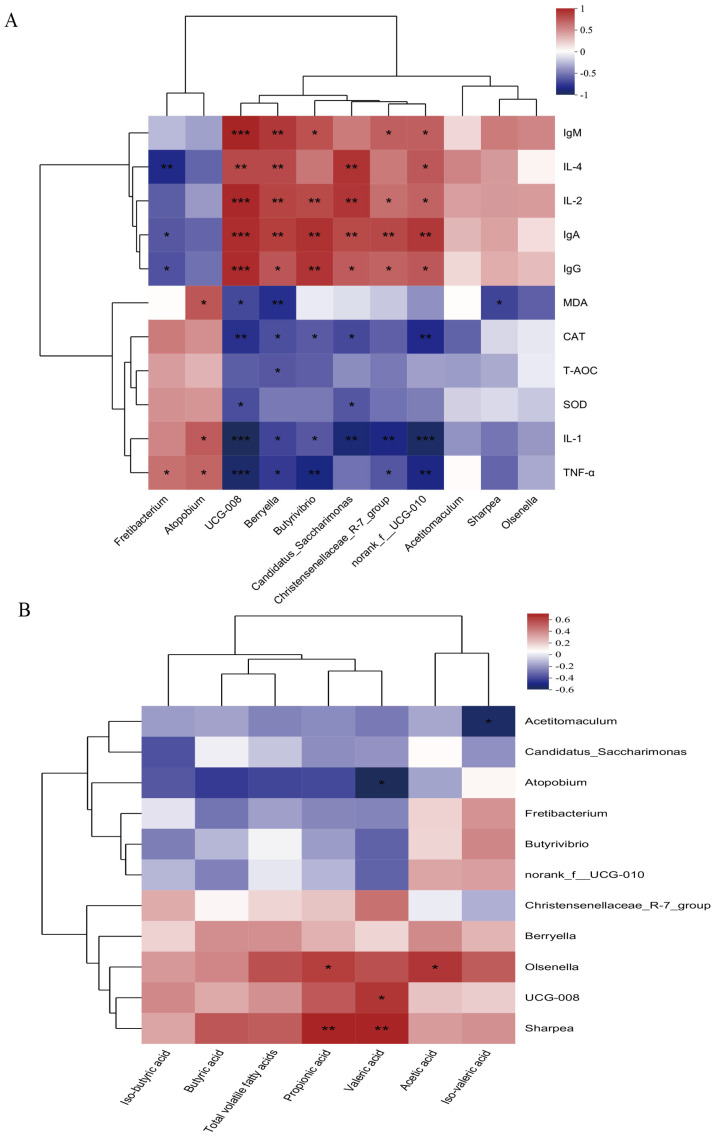
Spearman correlation analysis was used to quantify the covariation patterns at the genus level between the rumen microbiota and serum immune-related indicators as well as fermentation parameters. (**A**) Spearman correlation heatmap between rumen microbiota (genus level) and serum immune-related indicators, CAT: Catalase; T-AOC: Total Antioxidant Capacity; SOD: Superoxide Dismutase; MDA: Malondialdehyde; IL-1β: Interleukin-1 Beta; IL-2: Interleukin-2; IL-4: Interleukin-4; TNF-α: Tumor Necrosis Factor-Alpha; IgA/G/M: Immunoglobulin A/G/M. (**B**) Spearman correlation heatmap between rumen microbiota (genus level) and rumen fermentation parameters. Significance is indicated by asterisks: * *p* < 0.05, ** *p* < 0.01, *** *p* < 0.001.

Correlation analysis between rumen microbiota and host parameters (immune markers and VFAs) was performed using Spearman’s rank correlation coefficient. Both rows (microbial taxa) and columns (immune parameters and VFAs) were clustered using the average linkage method to generate a hierarchically structured heatmap. This analytical approach allowed us to identify significant associations between specific microbial communities and host physiological metrics while visualizing their overall relational patterns. According to the Spearman correlation heatmap, *Christensenellaceae_R-7_group*, *Candidatus_Saccharimonas*, and *Butyrivibrio* showed positive correlations with IgA, IgG, and IL-2, and negative correlations with IL-1β and TNF-α (*p* < 0.05) (Figure 4A). *UCG-008* was positively correlated with valeric acid, while *Olsenella* exhibited positive correlations with acetic acid and propionic acid. *Sharpea* was positively correlated with both propionic acid and valeric acid (*p* < 0.05) (Figure 4B).

## 4. Discussion

Starter feed is a type of functional feed specially formulated for pre-weaning lambs [21]. Its main functions include accelerating rumen development [22], making up for the nutritional gaps in ewe milk feeding [23], and helping lambs adapt to solid feed in advance, thus reducing the adverse effects caused by weaning stress [24]. The results of this experiment showed that initiating starter feed supplementation at 14 days of age significantly increased the rumen papilla width of lambs. This phenomenon can be attributed to the high content of fermentable carbohydrates in the starter feed [7]; these carbohydrates are fermented to produce VFAs, which provide sufficient energy for the development of rumen epithelial cells [6]. Therefore, we further studied the effects of different starter feed initiation ages on the expression of genes related to rumen epithelial growth. The results showed that initiating starter feed supplementation at 14 days of age upregulated the relative expression levels of *TGFβ1*, *IGFBP3*, and *IGFBP6*—genes closely related to rumen epithelial development. *IGFBP3* and *IGFBP6* play important roles in the growth and development of rumen epithelium by regulating the activities of *IGF-1* and *IGF-2* and promoting the morphological development of rumen tissue [25], which further confirms that the 14-day-old initiation group is the optimal choice. Previous studies have shown that *TGFβ1* can regulate ruminal papilla growth by promoting cell proliferation, and its expression level is significantly positively correlated with ruminal papilla length and width [26], indicating that increased papilla width can serve as an important indicator of rumen development [27]. A well-developed rumen significantly enhances the nutrient absorption capacity of ruminants, thereby promoting growth and health [1]. Consistent with these findings, we also observed that initiating starter feeding at 14 days of age improved BW and ADG in lambs, which aligns with previous research.

The concentration of NH_3_-N directly reflects the supply of available nitrogen sources for rumen microorganisms and is a key indicator for evaluating nitrogen utilization efficiency in ruminants [28]. The results of this trial showed that initiating starter feeding at 7 and 14 days of age significantly increased the NH_3_-N content in the rumen of lambs. This may be attributed to the fact that early supplementation at 7–14 days promoted rumen development and the colonization of protein-degrading bacteria, thereby enhancing the rate of dietary protein degradation in the rumen [29]. VFAs are the primary energy products of rumen fermentation [30]. Their total concentration and proportions serve as key indicators of fermentation patterns and internal environmental health, while also acting as critical regulators of microbial communities and host metabolism [31]. Acetate is the dominant volatile fatty acid produced by ruminal microbial fermentation, accounting for over 50% of the total metabolic energy derived from VFAs in the rumen [32,33]. Studies have shown that early initiation of starter feeding can increase acetate levels in the rumen. Elevated acetate concentration enhances energy supply and nutrient absorption efficiency in lambs, thereby promoting growth and development. Moreover, increases in acetate and propionate significantly stimulate the growth and development of ruminal papillae, expanding the absorptive surface area of the rumen epithelium and thereby improving nutrient absorption capacity [34]. Initiating starter feeding at 7 or 14 days of age resulted in ruminal elevations of acetate, propionate, and TVFA by 68.9–108.6%, 65.8–91.9%, and 51.8–58.1%, respectively, compared to initiation at 21 days, which is consistent with previous research. This effect may be attributed to the promotion of beneficial VFA-producing bacteria, such as *Christensenellaceae_R-7_group* and *Sharpea*, colonizing the rumen due to early starter feeding, thereby providing sufficient substrate for the production of acetate, propionate, and other VFAs. This finding also aligns with the earlier results indicating that starter feeding beginning at 14 days of age enhances rumen development and growth performance.

Early initiation of starter feeding can enhance the immune performance of lambs by improving intestinal development, upregulating immune-related gene expression, and increasing immunoglobulin levels and antioxidant capacity [4]. Compared with lambs that started starter feeding at 7 or 21 days of age, lambs in the 14-day initiation group showed increased serum concentrations of IgA, IgG, and IgM by 0.26–0.39 μg/mL, 0.69–1.15 μg/mL, and 0.49–0.86 μg/mL, respectively. This may be attributed to dietary transition challenges: lambs supplemented at 7 days may struggle to adapt rapidly from milk to solid feed, potentially impairing intestinal barrier function and altering immune regulation [35]. For instance, pro-inflammatory cytokines such as IL-1β and TNF-α can trigger inflammatory responses in various cells, leading to diarrhea and reduced growth performance in lambs [36,37]. In contrast, IL-2, IL-4, IgA, IgG, and IgM play essential roles in maintaining lamb health. IL-2 and IL-4 enhance immune responses primarily by modulating the activity of T and B cells, while IgA, IgG, and IgM contribute to humoral immunity by binding to pathogens [38,39]. Therefore, we speculate that initiating starter feeding at 14 days of age improves immune competence, reduces the incidence of diarrhea, and supports early healthy growth in lambs.

The rumen microbiota of lambs plays a vital role in nutrient metabolism [40], immune regulation [41], rumen health [42], and production performance [1]. Optimizing the rumen microbial community can significantly enhance lamb growth performance and health while reducing environmental impacts [1]. This study found that initiating starter feeding at 7 days of age significantly increased the relative abundance of cellulose-degrading bacteria in the rumen, such as *Christensenellaceae_R-7_group*, *Butyrivibrio*, and *Candidatus_Saccharimonas*. Previous studies have demonstrated their important functions in cellulose and protein digestion, which can improve the supply of VFAs and feed conversion efficiency. These bacteria also collectively form a barrier against pathogens, thereby enhancing the host’s immune capacity [43,44,45]. In addition, both *Christensenellaceae_R-7_group* and *Candidatus_Saccharimonas* are involved in amino acid metabolism and synthesis, further improving the animal’s nutrient digestion and absorption capabilities [46]. In a study on mouse gut microbiota, Xie et al. [47] reported that *Candidatus_Saccharimonas* plays an essential role in maintaining normal intestinal function. Moreover, an increase in its relative abundance has been shown to contribute to immune recovery in immunocompromised patients [48]. Therefore, we performed correlation analyses between these cellulose-degrading bacteria, VFAs, and immune parameters. Correlation analysis indicated statistically significant associations between differential rumen microbiota and VFAs. Furthermore, *Christensenellaceae_R-7_group*, *Butyrivibrio*, and *Candidatus_Saccharimonas* were positively correlated with IL-2, IL-4, IgA, IgG, and IgM. These findings indicate that early initiation of starter feeding can improve the ruminal environment, enhance gastrointestinal immunity, reduce the incidence of disease, and thereby promote growth performance in lambs—consistent with the results described above.

Similarly to the rumen microbiota, the intestinal microbiota of lambs plays a vital role in nutrient absorption, growth and development, immune function, and overall health [45]. The results of this trial demonstrated that initiating starter feeding at 14 days of age significantly increased the relative abundance of *Olsenella*, *Sharpea*, *UCG-008*, and Hornefia in the small intestinal microbiota of lambs. In contrast, supplementation starting at 21 days notably enhanced the relative abundance of *Berryella*, *Acinetobacter*, and *norank_f__Erysipelotrichaceae*. Interestingly, *Olsenella*, *Sharpea*, and *UCG-008* are strongly associated with intestinal immunity. *Olsenella*, a beneficial actinobacterium, is an important component of intestinal health and has been confirmed to correlate with reduced intestinal inflammation [49,50,51]. *Sharpea* is regarded as a key lactate-producing bacterium that can generate propionate via the acrylate pathway, thereby inhibiting methane production [52,53]. *UCG-008*, which belongs to the *Ruminococcaceae* family, has been shown to be involved in butyrate production and exhibits strong correlations with IgA, IgM, IgG, IL-1β, IL-2, and IL-6 [54]. Some studies suggest that lambs may have a critical window for immune system establishment between 0 and 42 days, during which *UCG-008* could serve as an immune marker for further research [55]. Qin et al. [56] also confirmed in a study on ducks with footpad dermatitis that *UCG-008* plays a significant role in inflammation regulation. On the other hand, *norank_f__Erysipelotrichaceae* has been found to potentially promote the secretion of inflammatory factors and impair intestinal mucosal permeability, thereby exacerbating the development of enteritis [57]. Wylensek et al. [58] discovered that *Berryella* facilitates the production of ornithine, citrulline, and arginine, playing an important role in host metabolism. Moreover, Berryella can metabolize dietary fiber to produce short-chain fatty acids (acetate, propionate, butyrate), which subsequently contribute to intestinal health and immune modulation. The results of this trial showed that lambs that received starter feed from 14 days of age showed significantly improved immune performance, which is largely consistent with previous findings. In conclusion, the 14-day initiation group, as the optimal supplementation strategy, can modulate the intestinal microbiota, promote the establishment of an intestinal immune barrier, and holds significant importance for early growth in lambs.

While this study provides comprehensive insights into the effects of starter feeding initiation age on suckling Hu lambs, it is important to acknowledge its limitations. The primary constraint is that the experimental period concluded at weaning (60 days of age). Consequently, the long-term persistence of the observed advantages in growth performance, rumen development, and microbial community structure into the post-weaning and finishing phases remains to be verified. Whether the optimal initiation window identified at 14 days confers enduring benefits on final BW, feed efficiency, and overall health after weaning is a critical question for both scientific understanding and practical application.

## 5. Conclusions

Initiating starter feeding at 14 days of age optimizes the growth performance, rumen development, immune function, and colonization of beneficial microorganisms in suckling Hu lambs. This timing is consistent with the natural maturation process of the gastrointestinal tract and microbial ecosystem of lambs, providing a practical strategy for improving lamb rearing effects. These research findings lay a scientific foundation for formulating precise early-life feeding management strategies to enhance the productivity of lambs and improve animal welfare. Therefore, initiating starter feeding at 14 days of age is recommended for suckling Hu lambs. Future studies will focus on validating these benefits in a long-term feeding trial to assess their persistence through the finishing phase.

## Figures and Tables

**Table 1:** Formula and Nutritional Composition of the Starter Diet.

Feed Formula	Content	Nutrient Level	Content
Corn	34.3%	Crude Protein	19%
Soybean meal	25%	Acid Detergent Fiber	14.8%
Wheat bran	5%	Neutral Detergent	22.1%
Limestone	2%	Calcium	1.1%
Corn straw	15%	Phosphorus	0.5%
Cottonseed meal	4%	Starch	30%
Corn germ meal	12.5%	Digestible Energy	2980 kJ/kg
Salt	0.5%		
Soda ash	0.2%		
Premix ^1^	1%		
Total	100%		

^1^ Premix provides the following mineral elements (mg·kg^−1^) and vitamins (IU·kg^−1^): S, 200; Fe, 25; Zn, 40; Cu, 8; Mn, 40; I, 0.3; Se, 0.2; Co, 0.1; VA, 940; VD, 111; VE, 20. DM, CP, NDF, Ca, and P were measured values, while the others were calculated values.

**Table 2:** Primer Sequence Information.

Gene	Primer Sequences (5′ to 3′)	Accession No.
*TGFβ1*	F:TGACCCACAGAGAGGAAATAGA R:AACCCGTTGATGTCCACTTGAA	NM_001009400.2
*IGFBP3*	F:TCAGCCTTGCGGCGTCTA R:TGTGGGCGAGGTGGGATT	NM_001159276.1
*IGFBP5*	F:GCTGAAGGCTGAGGCTGTGAA R:TCCCATACTTGTCCACGCACC	NM_001129733.1
*IGFBP6*	F:AGAGTAAGCCCCAAGCAG R:CACGGAGTCCAGATGTTT	NM_001134308.1
*IGF-1*	F:AAGATGCCAGTCACATCCTCC R:ATAAAAGCCCCTGTCTCCAC	NM_001009774.3
*IGF-1R*	F:AGCAAAGGCGACATAAACAC R:GGCTTCCTTGTAGTAGACGGT	XM_027957015.2
*β-actin*	F:GCTCTTCCAGCCGTCCTT R:TGAAGGTGGTCTCGTGAATGC	NM_205518.2

**Table 3:** Effects of Initiating Starter Feeding at Different Ages on Growth Performance of Suckling Lambs.

Days of Age	Treatment ^1^			SEM ^3^	*p*-Value		
	7 d Group	14 d Group	21 d Group		Treatment	Time	T × T ^2^
	BW ^3^, kg						
7	4.92	5.51	5.32	0.72	0.14	0.98	0.991
14	5.13	7.25	7.27	1.02	0.07	<0.01	<0.01
21	6.43	8.77	9.05	0.35	0.06	<0.01	<0.01
28	8.29	10.58	10.68	0.96	0.06	<0.01	<0.01
35	10.69	12.79	12.32	1.73	0.08	<0.01	<0.01
42	12.07	14.78	13.64	1.63	0.16	<0.01	0.01
49	12.78 ^b^	16.48 ^a^	15.12 ^a^	2.03	0.02	<0.01	0.02
60	14.72 ^b^	18.75 ^a^	16.70 ^a^	1.95	0.01	<0.01	0.01
	ADG ^3^, kg/d						
7–14	0.07	0.08	0.28	0.02	0.35	0.61	0.59
14–21	0.19	0.27	0.25	0.03	0.52	0.73	0.71
21–28	0.27	0.26	0.23	0.02	0.49	0.68	0.66
28–35	0.34 ^a^	0.32 ^a^	0.23 ^b^	0.03	0.02	<0.01	0.02
35–42	0.20	0.28	0.19	0.02	0.06	<0.01	0.03
42–49	0.25	0.24	0.21	0.04	0.68	0.82	0.79
49–60	0.28	0.32	0.23	0.05	0.13	0.22	0.21

In the same row, values with no letter or the same letter superscripts mean no significant difference (*p* > 0.05), while with different small letter superscripts mean significant difference (*p* < 0.05).^1^ The 7 d group represents lambs that started receiving starter feed at 7 days of age; the 14 d group represents those that started at 14 days of age; and the 21 d group represents those that started at 21 days of age. ^2^ T × T: Treatment × Time interaction effect. ^3^ BW: Body Weight; ADG: Average Daily Gain; SEM: Standard Error of the Mean.

**Table 4:** Effects of Initiating Starter Feeding at Different Ages on Rumen Fermentation Parameters of Lambs.

Item	Treatment ^1^			SEM ^3^	*p*-Value
	7 d Group	14 d Group	21 d Group		
pH	6.59	6.71	6.68	0.08	0.68
NH_3_-N ^2^, mg/dL	7.20 ^a^	9.09 ^a^	5.34 ^b^	0.56	0.01
Acetic acid, mmol/L	267.21 ^a^	216.21 ^a^	128.03 ^b^	29.35	0.01
Propionic acid, mmol/L	13.63 ^a^	15.89 ^a^	8.22 ^b^	1.97	0.02
Isobutyric acid, mmol/L	2.42	2.71	1.98	0.16	0.22
Butyric acid, mmol/L	11.78	10.53	10.41	1.47	0.35
Isovaleric acid, mmol/L	5.71	6.58	4.12	0.71	0.41
Valeric acid, mmol/L	4.18 ^ab^	6.85 ^a^	3.71 ^b^	0.69	0.01
TVFA ^2^, mmol/L	314.93 ^a^	258.77 ^a^	156.47 ^b^	9.42	0.01

In the same row, values with no letter or the same letter superscripts mean no significant difference (*p* > 0.05), while with different small letter superscripts mean significant difference (*p* < 0.05). ^1^ The 7 d group represents lambs that started receiving starter feed at 7 days of age; the 14 d group represents those that started at 14 days of age; and the 21 d group represents those that started at 21 days of age. ^2^ NH3-N: Ammonia Nitrogen; TVFA: Total Volatile Fatty Acids; ^3^ SEM: Standard Error of the Mean.

**Table 5:** Effects of Initiating Starter Feeding at Different Ages on Antioxidant Capacity and Serum Immunity of Lambs.

Item	Treatment ^1^			SEM ^5^	*p*-Value
	7 d Group	14 d Group	21 d Group		
CAT ^2^, μmol/mL	50.62	47.58	55.37	5.88	0.26
T-AOC ^2^, μmol/mL	0.15	0.11	0.11	0.01	0.26
SOD ^2^, U/mL	6.11	3.70	5.16	0.66	0.37
MDA ^2^, nmol/mL	0.17 ^a^	0.11 ^b^	0.12 ^b^	0.01	0.02
IL-1β ^3^, pg/mL	169.94 ^a^	93.41 ^b^	140.91 ^ab^	6.64	<0.01
IL-2 ^3^, pg/mL	649.02 ^b^	1241.29 ^a^	1017.77 ^ab^	49.77	<0.01
IL-4 ^3^, pg/mL	77.65 ^b^	128.03 ^a^	105.47 ^ab^	4.55	<0.01
TNF-α ^3^, pg/mL	67.63 ^a^	47.92 ^b^	68.99 ^a^	2.27	<0.01
IgA ^4^, μg/mL	0.63 ^b^	1.02 ^a^	0.76 ^ab^	0.04	<0.01
IgG ^4^, μg/mL	1.06 ^b^	2.21 ^a^	1.52 ^ab^	0.10	<0.01
IgM ^4^, μg/mL	1.43 ^b^	2.29 ^a^	1.80 ^ab^	0.08	<0.01

In the same row, values with no letter or the same letter superscripts mean no significant difference (*p* > 0.05), while with different small letter superscripts mean significant difference (*p* < 0.05). ^1^ The 7 d group represents lambs that started receiving starter feed at 7 days of age; the 14 d group represents those that started at 14 days of age; and the 21 d group represents those that started at 21 days of age. ^2^ CAT: Catalase; T-AOC: Total Antioxidant Capacity; SOD: Superoxide Dismutase; MDA: Malondialdehyde. ^3^ IL-1β: Interleukin-1 Beta; IL-2: Interleukin-2; IL-4: Interleukin-4; TNF-α: Tumor Necrosis Factor-Alpha. ^4^ IgA/G/M: Immunoglobulin A/G/M. ^5^ SEM: Standard Error of the Mean.

**Table 6:** Effects of Initiating Starter Feeding at Different Ages on the Expression of Gastrointestinal Epithelial Growth-Related Genes in Lambs.

Site	Item	Treatment ^1^			SEM ^3^	*p*-Value
		7 d Group	14 d Group	21 d Group		
Rumen	*TGFβ1* ^2^	1.01 ^b^	3.11 ^a^	2.78 ^ab^	0.21	<0.05
	*IGFBP3* ^2^	1.01	1.67	1.62	0.43	0.20
	*IGFBP5* ^2^	1.05	1.21	0.91	0.09	0.48
	*IGFBP6* ^2^	1.00	1.54	1.46	0.15	0.35
Duodenum	*IGF-1* ^2^	1.00	1.54	1.32	0.36	0.12
	*IGF-1R* ^2^	1.01	1.38	1.18	0.10	0.09

In the same row, values with no letter or the same letter superscripts mean no significant difference (*p* > 0.05), while with different small letter superscripts mean significant difference (*p* < 0.05). The same as below. ^1^ The 7 d group represents lambs that started receiving starter feed at 7 days of age; the 14 d group represents those that started at 14 days of age; and the 21 d group represents those that started at 21 days of age. The same naming convention applies consistently in the following tables. ^2^ *TGFβ1*: Transforming Growth Factor Beta 1; *IGFBP3*: Insulin-like Growth Factor Binding Protein 3; *IGFBP5*: Insulin-like Growth Factor Binding Protein 5; *IGFBP6*: Insulin-like Growth Factor Binding Protein 6; *IGF-1*: Insulin-like Growth Factor 1: *IGF-1R*: Insulin-like Growth Factor 1 Receptor. ^3^ SEM: Standard Error of the Mean.

**Table 7:** Effects of Initiating Starter Feeding at Different Ages on Gastrointestinal Tract Development of Lambs.

Site	Item	Treatment ^1^			SEM ^2^	*p*-Value
		7 d Group	14 d Group	21 d Group		
Rumen	Papilla length, μm	1750.61	1573.16	1687.21	96.78	0.43
	Papilla width, μm	697.33 ^a^	714.15 ^a^	598.33 ^b^	23.23	0.03
	Muscle thickness, μm	1108.33	1005.16	932.78	55.37	0.29
Duodenum	Villus height, μm	832.15	833.18	731.88	69.34	0.80
	Villus width, μm	163.22	150.31	147.19	28.31	0.15
	Crypt depth, μm	492.36	501.33	430.79	51.33	0.67
	Muscularis thickness, μm	337.11	381.72	360.72	28.14	0.24
	Villus height/Crypt depth ratio (V/C)	1.69	1.66	1.70	0.03	0.87
Jejunum	Villus height, μm	761.33 ^a^	612.56 ^b^	599.36 ^b^	47.13	<0.01
	Villus width, μm	143.51 ^a^	121.46 ^b^	117.75 ^b^	12.08	<0.01
	Crypt depth, μm	769.30 ^a^	551.65 ^b^	491.89 ^b^	37.97	<0.01
	Muscularis thickness, μm	201.49	198.17	203.33	21.28	0.35
	Villus height/Crypt depth ratio (V/C)	0.99	1.11	1.22	0.01	0.65

In the same row, values with no letter or the same letter superscripts mean no significant difference (*p* > 0.05), while with different small letter superscripts mean significant difference (*p* < 0.05). The same as below. ^1^ The 7 d group represents lambs that started receiving starter feed at 7 days of age; the 14 d group represents those that started at 14 days of age; and the 21 d group represents those that started at 21 days of age. The same naming convention applies consistently in the following tables. ^2^ SEM: Standard Error of the Mean.

## Data Availability

The original contributions presented in this study are included in the article. Further inquiries can be directed to the corresponding authors.
